# Comparative Genomic Studies of *Salmonella* Heidelberg Isolated From Chicken- and Turkey-Associated Farm Environmental Samples

**DOI:** 10.3389/fmicb.2018.01841

**Published:** 2018-08-10

**Authors:** Loïc Deblais, Benjamin Lorentz, Joy Scaria, Kakambi V. Nagaraja, Muhammad Nisar, Dale Lauer, Shauna Voss, Gireesh Rajashekara

**Affiliations:** ^1^Food Animal Health Research Program, Department of Veterinary Preventive Medicine, The Ohio State University, OARDC, Wooster, OH, United States; ^2^Department of Plant Pathology, The Ohio State University, OARDC, Wooster, OH, United States; ^3^Department of Veterinary and Biomedical Sciences, South Dakota State University, Brookings, SD, United States; ^4^Department of Veterinary and Biomedical Sciences, College of Veterinary Medicine, University of Minnesota, Saint Paul, MN, United States; ^5^Minnesota Poultry Testing Laboratory, University of Minnesota Veterinary Diagnostic Laboratory, Minnesota Board of Animal Health, Willmar, MN, United States

**Keywords:** *Salmonella* Heidelberg, poultry farms, whole genome, antibiotic-resistance genes, type IV secretion system, prophages

## Abstract

*Salmonella* is one of the leading causes of human foodborne gastroenteritis in the United States. In addition, *Salmonella* contributes to morbidity and mortality in livestock. The control of *Salmonella* is an increasing problematic issue in livestock production due to lack of effective control methods and the constant adaptation of *Salmonella* to new management practices, which is often related to horizontal acquisition of virulence or antibiotic resistance genes. *Salmonella enterica* serotype Heidelberg is one of the most commonly isolated serotypes in all poultry production systems in North America. Emergence and persistence of multi-drug resistant *Salmonella* Heidelberg isolates further impact the poultry production and public health. We hypothesized that distinct poultry production environments affect *Salmonella* genomic content, and by consequence its survival and virulence abilities. This study compared the genomic composition of *S.* Heidelberg isolated from environmental samples (19 chicken and 12 turkey isolates) of different breeder farms (16 chicken and 8 turkey farms) in the Midwest, United States. Whole genome comparison of 31 genomes using RAST and SEED identified differences in specific sub-systems in isolates between the chicken- and turkey-associated farm environmental samples. Genes associated with the type IV secretion system (*n* = 12) and conjugative transfer (*n* = 3) were absent in turkey farm isolates compared to the chicken ones (*p*-value < 0.01); Further, turkey farm isolates were enriched in prophage proteins (*n* = 53; *p*-value < 0.01). Complementary studies using PHASTER showed that prophages were all *Caudovirales* phages and were more represented in turkey environmental isolates than the chicken isolates. This study corroborates that isolates from distinct farm environment show differences in *S.* Heidelberg genome content related to horizontal transfer between bacteria or through viral infections. Complementary microbiome studies of these samples would provide critical insights on sources of these variations. Overall, our findings enhance the understanding of *Salmonella* genome plasticity and may aid in the development of future effective management practices to control *Salmonella*.

## Introduction

More than two million Americans get sick annually due to the consumption of food products contaminated with foodborne pathogens. Non-typhoidal *Salmonella* are among the top five enteric pathogens encountered in the United States. They are responsible for 11% of illnesses, 35% of hospitalizations, and 28% of deaths caused by foodborne pathogens in the United States, with an estimated cost of $3.6 billion.^1^ The United States is also the largest poultry producer in the world; however, poultry is the most common source of wide-scale salmonellosis outbreaks (Antunes et al., 2016). *Salmonella* intensively colonizes the intestinal track of chickens and turkeys, and in most cases contamination occurs during post-harvest manipulations of the carcass through several routes (evisceration, contaminated water, previously slaughtered *Salmonella*-positive flocks, equipment used in abattoirs, insects, or slaughterhouse personnel) (Wieczorek and Osek, 2015; Antunes et al., 2016). Since 1990, 53 live poultry-associated salmonellosis outbreaks were reported in the United States, causing 2,630 illnesses, 387 hospitalizations, and five deaths (Basler et al., 2016). It was estimated that *Salmonella* associated with poultry cost up to $695 million in public health (Batz et al., 2012). Among the approximately known 2,600 serotypes represented in this species, *Salmonella* *enterica* serotype Typhimurium and *S.* *enterica* serotype Enteritidis have the highest human incidence in the United States (Bugarel et al., 2017); however, several studies have reported *S. enterica* serotype Heidelberg as the most common serotype isolated in all breeder types in the United States and Canada, and throughout all levels of the production chain (Guerin et al., 2005; Zhang et al., 2005; Sivaramalingam et al., 2013). *S.* Heidelberg is most commonly isolated from egg containing products and poultry (Chittick et al., 2006). It was estimated that *S.* Heidelberg causes 84,000 illnesses per year in the United States, making it the sixth most common salmonellosis causal agent (Foley et al., 2011).

The poultry industry is constantly upgrading its management practices to prevent the introduction of *Salmonella* in poultry products (Dawkins, 2017; Mehdi et al., 2018). Foams, fumigant (formaldehyde), heat, and high-pressure treatments are used to disinfect the farm environment between flocks, while therapeutic and non-therapeutic agents (antibiotics, vaccines, feed additives, and antagonistic organisms) are used to control *Salmonella* in the flock (Mehdi et al., 2018). However, the effectiveness of these control methods decrease over time due to a constant adaptation of *Salmonella*. It has been pointed that the host, the farm environment where the birds are raised, the management practices, and the microbial population surrounding *Salmonella* can be at the origin of these adaptations (Foley et al., 2013). For example, control practices used in poultry industry influence the microbial community and, by consequence, the reservoir of antibiotic resistance genes (ARGs) that can be potentially transferred between bacteria (Nisar et al., 2017). It also has been found that *Salmonella* could persist within agricultural environments despite decontamination efforts, which could be the cause of newly emerging antimicrobial-resistant strains (Winfield and Groisman, 2003). An increase in antimicrobial-resistant *S.* Heidelberg was reported over the past years worldwide and cephalosporin resistant *Salmonella* are of concern (Hernandez et al., 2002; Dutil et al., 2010; Medeiros et al., 2011; Amand et al., 2013; Liakopoulos et al., 2016). Based on the National Antimicrobial Resistance Monitoring System for Enteric Bacteria (NARMS) 2014 report, cephalosporin resistant *S.* Heidelberg is a persistent problem in poultry and humans in the United States with 12.5% of *S.* Heidelberg isolates collected from retail chickens and 8.5% from humans in 2014 were cephalosporin resistant.^2^ It was suggested that the increase resistance of *Salmonella* to β-lactam such as cephalosporin was associated with the horizontal transfer of an IncA/C plasmid, which confers the resistance to several antimicrobials (Frye and Jackson, 2013). Therefore, it is imperative to understand how exogenous farming practices are affecting *Salmonella* virulence, antimicrobial resistance and survival in different poultry farm environment settings in order to device effective management strategies.

With the advent of next generation sequencing technologies, it is now possible to perform detailed whole genome studies to examine the gene composition and diversity in *Salmonella* isolated from different poultry farm environments (Strachan et al., 2015). In this study, the genomic content of two distinct populations of *S.* Heidelberg isolated from either chicken (*n* = 19) or turkey (*n* = 12) farm environments was analyzed. We hypothesize that *S.* Heidelberg isolates collected from the chicken- and turkey-associated farm environments would display unique differences in the genome content. The comparative genomic study of the isolates identified that 152 protein-encoding genes varied between *S.* Heidelberg from chicken- and turkey-associated farm environments. The majority of these genes are implicated into two sub-systems involved in horizontal gene transfers.

## Materials and Methods

### Farms, Sample Collection and Isolation of *S.* Heidelberg

**FIGURE 1:**
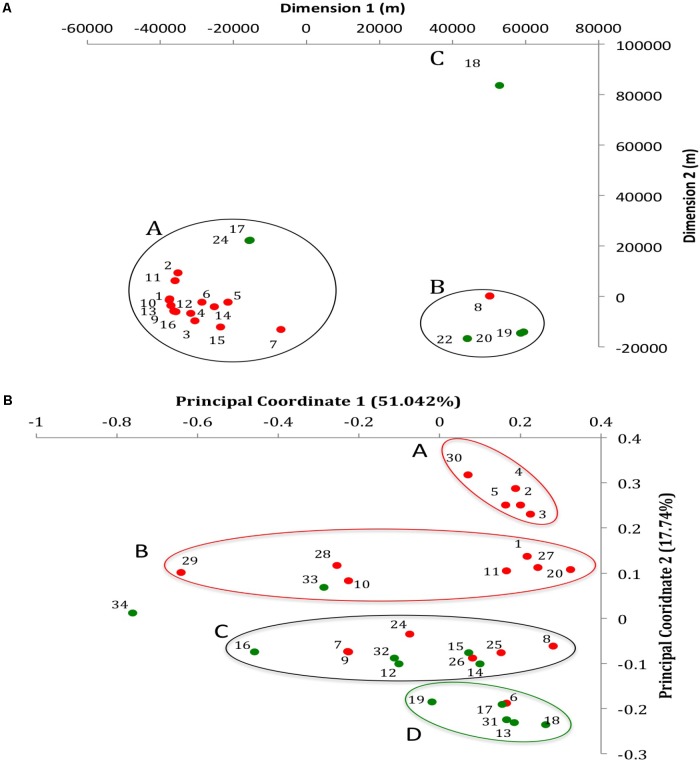
Geographic and whole genome differences between chicken and turkey isolates. **(A)** Multi-dimensional scaling plot of the 22 farms used to collect the *Salmonella* Heidelberg environmental isolates. Dimension 1 and 2 are in meters. Red dots are chicken farms while green dots are turkey farms. A, B, and C represent clusters based on a Wearn links value of 0.33 with smallest portion. Each dot is associated with a farm IDs as referred in **Supplementary Table S1**. **(B)** Principal coordinate analysis (PCoA) of the 31 isolates at the whole genome level using CAFE. PC1 (*X*-axis) explains 51.042% of variation while PC2 (Y-axis) explains 17.74% of variation. Red and green dots indicate whether the isolates come from chicken or turkey farms, respectively. Red circle: cluster principally composed of chicken farm isolates (clusters A and B); Green circle: cluster principally composed of turkey farm isolates (cluster D); Black circle: cluster composed of both turkey and chicken farm isolates (cluster C). Each dot is associated with an isolate ID as referred in **Supplementary Table S1**.

Samples from a total of 24 poultry farms (16 chicken and 8 turkey farms) from the Midwest, United States were used in this study. The geographic location of the farms where the samples originated is displayed in a multi-dimensional scaling plot (**Figure 1A**). Farms located within a radius of approximately 40 km from each other were considered from the same region (Wearn links of 0.33; smallest portion). Each farm housed turkey or chicken breeder flocks of a single age. The chicken flocks (broiler breeders) consisted of approximately 14,000 birds each. The turkey breeder flock numbers were more variable, between 10,000 and 18,000 birds per flock. Both chicken and turkey breeder farm operations have placed an increased emphasis on cleaning and disinfection in order to reduce *Salmonella* populations. The chicken farms operates with additional labor to complete the cleaning of buildings and equipment, and used multiple disinfectants prior to the application of formaldehyde. The turkey farms had dedicated cleaning crews and targeted 3 weeks down time between flocks. Building ceilings, walls and nests were washed before the litter was pushed out. A second washing of floors was performed followed by disinfectants before a final application of formaldehyde. Drinkers, feeders, and nest pads were cleaned outside.

The farm environmental samples were collected under the supervision of the Minnesota Board of Animal Health between April and July 2015 as part of “National Poultry Improvement Plan” (NPIP) *Salmonella* monitoring programs. A total of 29 *S.* Heidelberg isolates were collected from environmental booties (19 from chicken and 10 from turkey farms) and two *S.* Heidelberg isolates were collected from hatchery debris in turkey farms. Details concerning the isolates and farms are displayed the **Supplementary Table S1**.

Collection of the samples was performed as described in the NPIP.^3^ Briefly, absorbable fabric shoe covers (booties) were used inside the farms and exposed to the surface of the floor litters over a distance of 305 m (1000 feet). Hatcher fluff samples were collected by placing fluff material from the floor of the hatcher directly into a sterile bag. Both environmental booty and hatcher fluff samples were enriched with Tetrathionate enrichment broth at a ratio of 1:10 (sample to enrichment) at 40°C for 20 h. Samples were then plated on selective agar plates (brilliant green with novobiocin, xylose lysine tergitol-4, and Miller-Mallinson) and incubated at 40°C for 20 h. *Salmonella*-like colonies were transferred to triple sugar iron agar slants and incubated at 40°C for 20 h. If the hatcher fluff samples were negative for *Salmonella* after the initial Tetrathionate enrichment, samples were retained for a Delayed Secondary Enrichment procedure. All suspect *Salmonella* isolates were serogrouped and serotyped using traditional plate and tube agglutination tests.

The identity of *Salmonella* isolates was further confirmed using a polymerase chain reaction (PCR) (Nisar et al., 2017). The DNA of the pure colonies was extracted using a QIAamp DNA mini kit (Qiagen, Valencia, CA, United States) and then quantified using a Nano-Drop ND-2000 spectrophotometer (Thermo Fisher Scientific, Waltham, MA, United States). One set of primers specific to the *Salmonella* genus (Target: OMPC; forward: ATCGCTGACTTATGCAATCG; reverse: CGGGTTGCGTTATAGGTCTG; amplicon length = 204 bp) and another set specific to the Heidelberg serotype (Target: ACF69659; forward: TGTTTGGAGCATCATCAGAA; reverse: GCTCAACATAAGGGAAGCAA; amplicon length = 216 bp) were used to confirm the identity of the isolates (Alvarez et al., 2004; Park and Ricke, 2015; Nisar et al., 2017). PCR amplification was performed in Eppendorf EP Mastercycler S machine with an initial denaturation step at 95°C for 10 min, followed by 35 cycles of denaturation at 94°C for 1 min, annealing at 50°C for 1 min, extension at 72°C for 1 min. Then a final extension was performed at 72°C for 10 min before storing samples at 4°C. PCR products were visualized by gel electrophoresis under UV light in 1.2% agarose gel (Park and Ricke, 2015; Nisar et al., 2017).

### DNA Extraction and Whole Genome Sequencing

The DNA from each *S.* Heidelberg isolate was isolated from 1 ml of grown cultures using E.Z.N.A.^®^ Bacterial DNA Kit (Omega Bio-tek, Norcross, GA, United States). The concentrations of genomic DNA samples were measured using Qubit Fluorometer 3.0 (Invitrogen, Carlsbad, CA, United States) and the concentration was adjusted to 0.2 ng/μl. After normalization, sequencing libraries were prepared using Nextera XT DNA Sample Prep Kit (Illumina Inc., San Diego, CA, United States). Tagmentation of samples using 1 ng of template was conducted according to Nextera XT DNA library Prep manufacturer’s protocol, followed by PCR amplification of the library. Indexing was done using Nextera XT Index 1 Primers (N7XX) from the Nextera XT Index kit (FC131-1001). Briefly, PCR amplification was performed in Veriti 96-well Thermal Cycler machine (Thermo Fisher Scientific) with an initial denaturation step at 95°C for 10 min, followed by 12 cycles of denaturation at 95°C for 10 s, annealing at 55°C for 30 s, extension at 72°C for 30 s. Then a final extension was performed at 72°C for 5 min before storing samples at 10°C. PCR products were then cleaned using Agencourt AMpure XP beads (Beckman Coulter). Purified products were normalized using library normalization protocol suggested by Illumina. Equal volumes of normalized libraries were pooled together and diluted in hybridization buffer. The pooled libraries were heat denatured and spiked with 5% of the Illumina PhiX control DNA prior to loading the sequencer. Illumina paired-end sequencing was performed on the MiSeq platform using a 2× 250 paired-end sequencing chemistry. The raw data files were de-multiplexed and converted to FASTQ files using Casava v.1.8.2. (Illumina, Inc., San Diego, CA, United States).

### Bioinformatic Analysis

After sequencing, a quality control of the raw reads was performed using FastQC (Babraham Bioinformatics, Cambridge, MA, United States). Only nucleotides with a base sequence quality whose median quality score above 25 and whose lower quartile median quality score above 10 were used for further analysis. The reads were trimmed with BBDuk (DOE Joint Genome Institute, Walnut Creek, CA, United States) using an average quality cutoff of 10. Reads were assembled using SPADEs (SPBU, Saint Petersburg, Russia) with a custom k-mer values of 31, 61, 99, 101, and121 mer lengths. Finally, the coverage of the assembled genomes was evaluated with BBMap (DOE Joint Genome Institute, Walnut Creek, CA, United States).

Whole genome diversity between isolates was studied using a pairwise distance matrix based on a Manhattan measurement and neighbor joining method. PCoA was generated using aCcelerated Alignment-FrEe sequence analysis (CAFE) software^4^ (Lu et al., 2017). In order to study the gene content between genomes, the 31 genomes were annotated with RAST server,^5^ using *S.* Heidelberg strain SL 476 (taxonomical ID 454169) as reference genome (Aziz et al., 2008). Comparative genomic analyses were performed at the function role level based on data generated in RAST and clustered into specific sub-systems using SEED^6^ (Aziz et al., 2008; Overbeek et al., 2014). ARGs profile studies were performed using CARD^7^ and ARDB^8^ (Liu and Pop, 2009; Jia et al., 2017). PHASTER was used to study the prophage population in each genome^9^ (Arndt et al., 2016). Only the prophage identified as “intact” (score <90) was selected for the analysis and interpretation of the data. The present or absence of a protein-encoding gene in a given isolate was labeled one or zero, respectively. The distribution of each protein-encoding gene was compared between chicken and turkey farm isolates for each data set generated (Merhej et al., 2009).

### Statistical Analysis

The clusterization of the farms based on their geographic proximity was performed using a multi-dimensional scaling plot combined with a Wearn links value of 0.33 with smallest portion. The distribution of protein-encoding genes was compared between the chicken- and turkey-associated farm environmental samples using a one-way analysis of variance (ANOVA), followed by Student’s *t*-test with JMP PRO 12 software (Cary, NC 27513) (Merhej et al., 2009). A *p*-value of ≤0.01 was considered as significant.

### Accession Numbers

This Whole Genome Shotgun project has been deposited in NCBI GenBank under the Bioproject PRJNA417775.^10^ The accession numbers of each genome are displayed in **Supplementary Table S1**. Raw sequence reads have been deposited in the NCBI sequence read archive (SRA; SRP126070).

## Results

### *S.* Heidelberg Isolates From Chicken and Turkey Farm Environments Displayed Differences at the Whole Genome Level

The average coverage depth for all 31 genomes was 82.30×, which exceeded the minimum coverage of 60× recommended for *de novo* genome assembly (Pightling et al., 2014). The average genome size was 4,822,758 ± 88,213 base pairs and 4,880,074 ± 76,006 base pairs for the chicken and turkey farm isolates, respectively. Additional information concerning the assembled genomes of the 31 *S.* Heidelberg isolates are available in the NCBI website under the Bioproject accession PRJNA417775. The accession numbers of each genome are displayed in **Supplementary Table S1**. Further, a whole genome comparison of the 31 genomes was performed *de novo* based on a pairwise-distance matrix. Results were displayed using a principal coordinate analysis (PCoA) plot (**Figure 1B**). Three isolates (T_NS034, C_NS029, and T_NS016) displayed strong spatial differences compared to the other isolates (*n* = 28) based on the principal coordinate 1 (PC1), which explained 51.042% of the variation observed between isolates. On the other hand PC2, which explained 17.74% variation between isolates, separated 12 chicken farms isolates from the turkey farms isolates. Out of these 12 isolates, two clusters (cluster A and B; *n* = 7 and 5) were formed based on PC2 as well. Another cluster mostly composed of turkey isolates (cluster D; *n* = 5) displayed strong spatial profile differences with the two chicken clusters. And one heterogeneous cluster (cluster C) composed of both chicken (*n* = 6) and turkey (*n* = 5) isolates was located in between these homogeneous clusters. These data are supported by our earlier studies using pulsed-field gel electrophoresis (Nisar et al., 2017), which also indicated that most of the isolates from similar farm environment type displayed closely related fingerprint profile (87% similarity for chicken farm isolates and 88% similarity for turkey farm isolates). Further, to confirm whether these observations were potentially caused by the geographic location of the farms, a multi-dimensional scaling plot of the spatial location of the farms between each other was performed (**Figure 1A**). Twenty-nine environmental samples (10 isolates from turkey and 19 isolates from chicken farms) were collected from 22 farms (six turkey and 16 chicken farms). Seventeen farms were clustered within 25 kilometers radius (cluster A), which was approximately 70 km away from the cluster B (*n* = 4). The cluster A was mostly composed of chicken farms (88%) while the cluster B was mostly composed of turkey farms (75%). Only one turkey farm (farm 18 where T_NS-013 was isolated) was located more than 80 km away from other farms. Despite these spatial variations between farm locations, no correlation between the location and the whole genome differences observed in the PCoA were detected (**Figure 1B**). Given the source of the birds diverged between breeder farms, we are excluding the possibility that turkey and chicken isolates came from only two distinct *S.* Heidelberg ancestors.

### Distinct Gene Content Differences Were Observed in *S.* Heidelberg Isolated From Chicken- and Turkey-Associated Farm Environments

After annotation of the 31 genomes using the *S.* Heidelberg strain SL 476 reference genome, an average of 4800 ± 95.59 protein-encoding genes per genome were obtained compared to the 4,884 genes expected based on the reference genome in the Joint Genome Institute Integrated Microbial & Microbiome Genome samples database.^11^ No significant differences were observed between the average number of proteins-encoding genes found in the 19 genomes from chicken environmental farm isolates (4770.58 ± 93.94 genes) and in the 12 genomes from turkey environmental farm isolates (4843.25 ± 92.58 genes). These results suggested that the differences described above in the **Figure 1B** may be explained by differences in specific function role (role that a gene or gene product may play in the operation of a cell^12^) or sub-systems (collection of functional roles that are associated to each other in a system^13^).

After processing of the genome using Rapid Annotation using Subsystem Technology (RAST), 4,346 function roles were predicted among the 31 genomes studied. These function roles were clustered into sub-systems (*n* = 26) using SEED based on RAST annotations. Of the 26 sub-systems representing all *S.* Heidelberg genomes in this study, eight sub-systems displayed significant differences in the amount of function roles observed between the two production systems (*p*-value <0.01; **Table 1**). Protein-encoding genes associated with “phages, prophages, transposable elements and plasmids,” “stress response,” and “iron acquisition and metabolism” sub-systems were significantly higher in the turkey farm isolates compared to the chicken farm isolates (*p*-value <0.01). On the other hand, protein-encoding genes associated with “regulation and cell signaling,” “phosphorus metabolism,” “respiration,” “motility and chemotaxis,” and “nitrogen metabolism” sub-systems were significantly higher in the chicken farm isolates compared to the turkey farm isolates (*p*-value <0.01). These differences were not correlated with the farm locations (**Figure 1A**), which support that the environment might affect the gene content of specific sub-systems in *Salmonella*.

**Table 1:** Average protein-encoding genes per genome at the sub-system level.^∗^

Sub-systems	C_env_^a^	T_env_^a^	(T_env_–C_env_)^b^
Phages, prophages, transposable elements, plasmids	52.9 ± 2.6^B^	85.8 ± 2.3^A^	32.86
Membrane transport	262.2 ± 17.7^A^	264.4 ± 18^A^	2.26
Protein metabolism	271.2 ± 1.7^B^	273.4 ± 2.4^A^	2.26
Virulence, disease and defense	98.2 ± 4^B^	100.4 ± 5.8^B^	2.26
Stress response	177.4 ± 0.7^B^	179.3 ± 0.5^A^	1.96
Iron acquisition and metabolism	27.1 ± 0.2^B^	28 ± 0^A^	0.95
Cofactors, vitamins, prosthetic groups, pigments	306.9 ± 1.5^B^	307.5 ± 1.4^B^	0.61
Nucleosides and nucleotides	111.2 ± 1.4^A^	111.6 ± 1.5^A^	0.37
Dormancy and sporulation	3.1 ± 0.3^B^	3.3 ± 0.5^B^	0.23
Metabolism of aromatic compounds	39.4 ± 1^A^	39.5 ± 0.9^A^	0.08
Fatty Acids, Lipids, and Isoprenoids	130.2 ± 0.6^A^	130.3 ± 0.6^A^	0.04
RNA metabolism	259.1 ± 0.2^A^	259.1 ± 0.3^A^	0.03
Secondary metabolism	4 ± 0^A^	4 ± 0^A^	0
Potassium metabolism	28 ± 0^B^	28 ± 0^B^	0
Miscellaneous	56.1 ± 0.2^A^	56 ± 0^A^	-0.05
Sulfur metabolism	38.9 ± 0.6^B^	38.8 ± 0.4^B^	-0.11
Cell division and cell cycle	38.6 ± 1.2^A^	38.3 ± 0.9^A^	-0.33
Amino acids and derivatives	438.6 ± 1.2^A^	437.9 ± 1.6^A^	-0.66
Nitrogen metabolism	74.1 ± 0.6^A^	73.2 ± 0.4^B^	-0.94
Motility and chemotaxis	79.1 ± 0.7^A^	78 ± 0^B^	-1.11
DNA metabolism	134.8 ± 3.2^A^	133.4 ± 2.8^A^	-1.37
Phosphorus metabolism	51.7 ± 0.7^A^	50 ± 0^B^	-1.68
Respiration	220.6 ± 1.3^A^	218.9 ± 0.9^B^	-1.71
Carbohydrates	704.9 ± 3^A^	703 ± 1^B^	-1.89
Cell wall and capsule	279.8 ± 1.3^A^	277.9 ± 1.9^B^	-1.93
Regulation and cell signaling	141.8 ± 1.8^A^	139.1 ± 0.9^B^	-2.76

*^∗^Collection of functional roles that are associated to each other in a system.*

*^a^Average of protein-encoding genes per genome for a given sub-system and its standard deviation. Letters associated with each number indicate which statistical group the value belongs to for the selected sub-system (Student t-test; oxp-value <0.01).^b^Protein-encoding gene differences between T_env_ and C_env_. C_env_, chicken environmental isolates (n = 19). T_env_, turkey environmental isolates (n = 12).*

**FIGURE 2:**
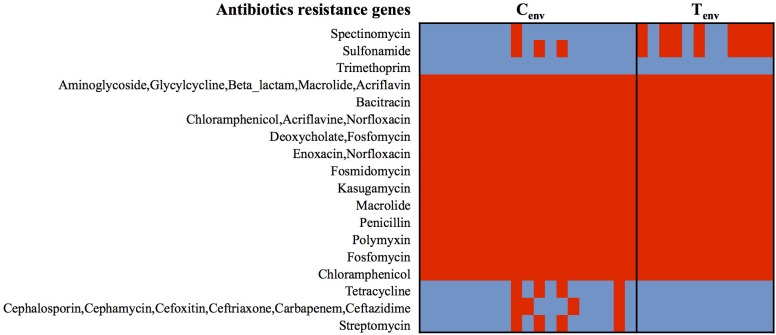
Heatmap of genes detected in *S.* Heidelberg isolates associated with antibiotic-resistance. Red cell: protein-encoding gene detected; Blue cell: protein-encoding gene missing. C_env_, chicken environmental isolates (*n* = 19); T_env_, turkey environmental isolates (*n* = 12).

At the protein function role level, less than 3% of protein-encoding genes were inconsistently detected within the isolates from a same farm environment type (protein-encoding genes detected between 30 and 70% of the isolates in each farm environment type were considered as inconsistent); while up to 13.53% of protein-encoding genes were inconsistent between chicken- and turkey-associated farm environmental isolates and 3.5% of them were significantly different (*n* = 152; *p*-value <0.01). Prophages and type IV secretion systems (T4SS) protein-encoding genes displayed high gene content variability between chicken and turkey isolates (**Supplementary Figure S1**). A total of 53 protein-encoding genes related to prophage functions were either only detected in turkey farm isolates or significantly higher in turkey farm isolates compared to the chicken farm isolates, which represented 50.5% (53/105) of the protein-encoding genes (*p*-value <0.01; **Supplementary Figure S1**). These results suggest that viral infections could occur at higher rate in a turkey farm setting than in chicken farms, and prophages could be a source of genomic alterations for the turkey isolates. On the other hand, all 12 T4SS protein-encoding genes were detected only in chicken farm isolates, suggesting that only chicken isolates may possess functional T4SS and this might be a mechanism for exchanging genetic material among chicken farm isolates (**Supplementary Figure S1**). Twenty-nine percent of protein-encoding genes, either significantly higher or only detected in one of the farm environmental type, had no biological functions identified or were designated as “hypothetical protein” (**Supplementary Figure S1**). The remaining protein-encoding genes identified in both chicken- and turkey-associated farm environmental isolates were related to stress responses (nutrient deficiencies, iron uptake, and temperatures), replication, and transcription mechanisms (**Supplementary Figure S1**). Further, general ARGs profile studies performed with the Comprehensive Antibiotic Resistant Database (CARD) and the Antibiotic Resistance Gene Database (ARDB) showed that all 31 isolates possessed genes associated with the resistance to 12 different antibacterial agents (aminoglycoside, bacitracin, fosfomycin, kasugamycin/macrolides, penicillin, chloramphenicol, enoxacin/norfloxacin, fosmidomycin, deoxycholic acid, and polymyxin; **Figure 2**). Twenty-one percent of the environmental isolates from chicken farms (*n* = 4/19) carried genes associated with the resistance to cephalosporin, tetracycline, and streptomycin; while 67% of the isolates from turkey farms (*n* = 8/12) carried genes associated with the resistance to spectinomycin and sulfonamide. Most of these predicted antibiotic resistant genes were detected from the same isolates; nevertheless, the variations in antibiotic resistance associated genes per isolate did not explain the variation observed in the **Figure 1B** and were not influenced by the geographic location of the farms (**Figure 1A**). Despite the consistent predictions of antibiotic resistance genes between CARD and ARBD, the antimicrobial susceptibility assay performed in our earlier studies with the same isolates than those used for sequencing showed some discrepancies with the *in silica* prediction concerning the resistance to chloramphenicol, tetracycline, streptomycin, and ceftriaxone (Nisar et al., 2017). On the other hand, the *in silica* predictions were concordant for the resistance to ceftriaxone/cefoxitin/ceftiofur, ciprofloxacin, nalidixic acid, and sulfonamide.

### Prophage DNA Varied Between Turkey and Chicken Farm Isolates

**FIGURE 3:**
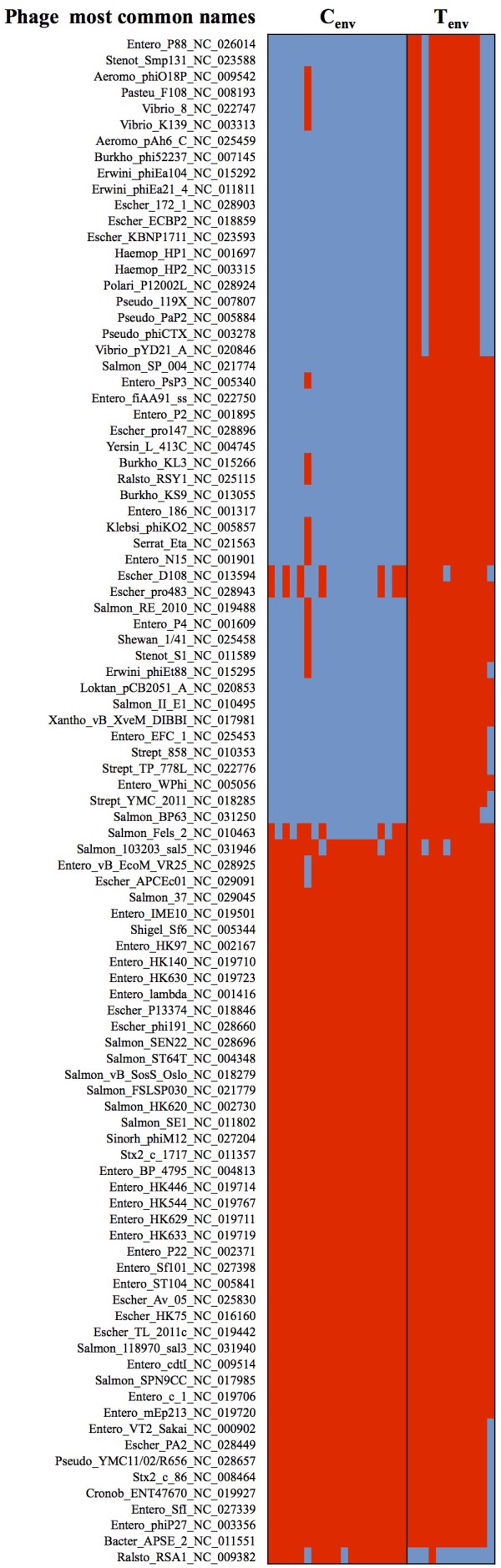
Heatmap of prophages showing significant differences between chicken- and turkey-associated farm environmental isolates. Red cell: prophage sequence detected; Blue cell: prophage sequence missing. C_env_, chicken environmental isolates (*n* = 19). T_env_, turkey environmental isolates (*n* = 12). Phage most common name: (host where the phage was first discovered)_(phage name)_(NCBI accession number). *N* = 122 prophages.

Studies of the prophage diversity in each isolate also identified distinct profiles between the two sets of environmental isolates. Based on the Phage Search Tool – Enhanced Released (PHASTER) outputs, a total of 4,296 prophage parts belonging to 272 different prophages were detected throughout the 31 genomes with high confidence (completeness labeled as “intact”; score >90). More than 50% of the prophage parts were identified as “integrase.” A total of 236 prophage parts were detected in the turkey environmental farm isolates with an average of 188 ± 25 prophage parts per genome, and 133 prophage parts in the chicken environmental farm isolates with an average of 100 ± 14 prophage parts per genome. These two sets of environmental isolates had 118 prophage parts in common and 88 prophage parts significantly more abundant in turkey environmental farms isolates compared to chicken environmental farm isolates. Further 50 out of the 88 prophage parts were not detected in chicken environmental farm isolates, while only two prophage parts were significantly higher in chicken environmental farm isolates compared to the turkey environmental farm isolates, and only one was unique to the chicken environmental farm isolates (**Figure 3**). All 51 (50 from turkey isolates + one from chicken isolates) unique prophage parts were dsDNA viruses belonging to several families of the *Caudovirales* order. These results suggest that in a turkey farm environment prophage infections are more common than the chicken farm environment.

## Discussion

Poultry meat is a major source of protein in the United States and worldwide, however, it also represents a significant risk for wide-scale foodborne gastroenteritis outbreaks. Bacteria such as *Salmonella* are able to gain or lose genetic elements that allow them to survive in hostile environments and render management practices less or not effective (Davies, 2007; Davies and Davies, 2010). In this study, we compared the genome composition of *S*. Heidelberg isolates obtained from two distinct poultry production environments (turkey and chicken breeder farms) to determine whether *S.* Heidelberg isolates will display specific gene content differences between the two poultry farm environments studied.

Whole genome comparison data showed that 63% of chicken and 42% of turkey farm isolates were clustered by farm environmental type. Further, out of the 26 sub-systems characterizing the *S.* Heidelberg genome, eight sub-systems showed gene content differences between chicken- and turkey-associated farm environmental isolates. These differences observed at the genome level and at the protein-encoding genes’ level within and between the two farm environmental types were not correlated with the geographic distribution of the farms, suggesting that to some extent farm environment might have an impact on *S.* Heidelberg genome content and its predicted functionality. Differences observed at the sub-system level might lead to potential adaptations for survival and expression of new virulence features. For example, five genes encoding proteins (GroEL, GroES, SopE, SfmH, and COX) directly or indirectly involved in *Salmonella* virulence and survival in macrophage cells were significantly more represented or only detected in turkey environmental isolates (**Supplementary Figure S1**; Buchmeier and Heffron, 1990; Uchiya and Nikai, 2004; Moreau, 2015).

Horizontal transfer of genetic content is a predominant adaptation feature for bacteria. These exchanges often involve ARGs as well as virulence and survival-related genes (Huddleston, 2014). Fifteen genes involved in the T4SS and conjugative transfer were detected in the chicken farm isolates but not in the turkey farm isolates. The T4SS is composed of 12 structural proteins, which all of them were detected in the chicken farm isolates but were missing in turkey farm isolates, suggesting that only *S.* Heidelberg isolated from chicken farms may have a functional T4SS (Wallden et al., 2010). This findings would suggest that chicken isolates might be more likely to acquire foreign DNA than the turkey isolates through conjugative transfer using the T4SS (Zechner et al., 2012; Juhas, 2015). Also, three conjugative transfer proteins-encoding genes (*traG, traR*, and *kikA*) were detected only in chicken isolates (**Supplementary Figure S1**). These proteins are associated with the IncQ-related and IncN plasmid groups (Bönemann et al., 2006). The IncQ-related plasmid group is a broad-host-range transmissible plasmid linked to resistance against quinolones, an antibiotic used in poultry against *Salmonella* (Bönemann et al., 2006). On the other hand, the IncN plasmid group was previously identified as a potential reservoir for extended-spectrum β-lactamase (ESBL) genes (Börjesson et al., 2016). Several studies, in concordance with ours, reported that these two types of plasmid and the ESBL genes are highly prevalent in the *Enterobacteriaceae* family from chicken farm isolates (Rawlings and Tietze, 2001). Nevertheless, we acknowledge that these results were based on *in silica* predictions; therefore, further analysis on the antimicrobial resistance phenotypes should be performed to confirm these predictions.

For a long time, horizontal transfers were known to be mostly caused by plasmid mediated conjugative transfer and by transposons; however, recent studies proposed transduction as a greater driving force for bacterial evolution than expected due to the broad diversity of bacteriophages that could be found in bacterial genome in a lysogenic stage (Balcazar, 2014; Shousha et al., 2015; Keen et al., 2017). Our study detected intact prophage parts from 272 different prophages among the 31 genomes with distinct prophage diversity profile based on the farm environmental type. The number of prophage parts identified in turkey environmental isolates was 1.77-fold higher compared to the chicken environmental isolates, suggesting that viral transduction might be frequently occurring in bacteria isolated from the turkey farm environment and turkey production environment may facilitate this process readily. The origin of these differences remains unclear; complementary information concerning the farm history and management practices could provide decisive details that might explain the drastic difference in prophage-related genes observed between the two farm environmental types. Previous studies have indicated that the use of antimicrobials might facilitate the intra- and inter-species horizontal gene transfers by the induction of phage-mediated gene transfer (Allen et al., 2011; Modi et al., 2013; Balcazar, 2014; Bearson and Brunelle, 2015). Prolonged exposure of *S*. Typhimurium isolates to carbadox led to the induction of transducible phages containing sections of the bacterial genome (Bearson et al., 2014). Moreover, the virome of antibiotic treated mice was highly enriched in ARGs compared to the untreated ones, and this enriched virome was more likely to transmit ARGs to a new bacterial population (Modi et al., 2013). This phenomenon was also identified in *Enterobacteriaceae* and *Salmonella* (Colavecchio et al., 2017). Some phages were able to transduce ARGs from the *S.* Typhimurium DT104 strain to other bacteria (Schmieger and Schicklmaier, 1999). Given that *S.* Heidelberg isolates in our study displayed a broad diversity of phage-related genes, the risk of phage horizontal transfers due to the use of various antimicrobials such as carbadox and fluoroquinolone in turkey farms may be higher.

## Conclusion

In conclusion, our data showed significant differences in distinct sub-systems in *S.* Heidelberg isolated from two different poultry production environments. The genetic materials involved in conjugative and phage horizontal transfer were differentially represented in chicken and turkey farm isolates, which might contribute to *Salmonella* genome plasticity, and thereby emergence of antibiotic resistance, survival and virulence abilities. Conjugative and phage horizontal transfers are sources of bacterial genome evolution (Frost et al., 2005); therefore, future studies on the global microflora genomic composition from both chicken and turkey production systems may shed light on the origin of these differences observed in the *Salmonella* genome and identify potential agents/pathways associated with *Salmonella* survival in different poultry farm environment.

## Ethics Statement

The *Salmonella* isolates in this study were isolated and identified by Laboratory of Board of Animal Health in Minnesota. The Laboratory of Board of Animal Health meets the requirements of CFR147.52, which allowed the collection of samples by following the National Poultry Improvement Plan (NPIP), USDA guidelines. No Institutional Animal Care and Use Committee (IACUC) approval was required to conduct this study. Permission from owners was obtained according to NPIP program standards to collect the samples. The university personal was not involved in any sample collection. No field permits were required to obtain these isolates.

## Author Contributions

GR and LD conceived and designed the experiment and wrote the manuscript. KVN, DL, SV, and MN collected the samples and isolated *S.* Heidelberg. JS performed the sequencing of the *S.* Heidelberg isolates. LD and BL performed bioinformatics analysis of the whole genome data.

## Conflict of Interest Statement

The authors declare that the research was conducted in the absence of any commercial or financial relationships that could be construed as a potential conflict of interest.

## Footnotes

1 https://www.ers.usda.gov/data-products/cost-estimates-of-foodborne-illnesses/

2 https://www.fda.gov/AnimalVeterinary/SafetyHealth/AntimicrobialResistance/NationalAntimicrobialResistanceMonitoringSystem/ucm059103.htm

3 https://www.poultryimprovement.org/documents/ProgramStandardsAugust2014.pdf

4 https://github.com/younglululu/CAFE

5 http://rast.nmpdr.org

6 http://pubseed.theseed.org/

7 https://card.mcmaster.ca

8 https://ardb.cbcb.umd.edu

9 http://phaster.ca

10 http://www.ncbi.nlm.nih.gov/bioproject/417775

11 https://img.jgi.doe.gov/cgi-bin/m/main.cgi?section=TaxonDetail&page=taxonDetail&taxon_oid=642555156

12 http://www.theseed.org/wiki/Glossary#Functional_role

13 http://www.theseed.org/wiki/SEED_Viewer_Manual/Subsystems

## Supplementary Material

FIGURE S1 Heatmap of protein-encoding genes showing significant differences between production systems at the function level. Heatmap: the gradient of red is proportional to the number of protein-encoding genes detected for a specific function role, and white cells are for protein-encoding genes not detected. Numbers in each cell represent the amounts of protein-encoding genes with the designated function role. C_env_: chicken environmental isolates (*n* = 19); T_env_: turkey environmental isolates (*n* = 12). *N* = 152 protein-encoding genes.

TABLE S1 *Salmonella* Heidelberg isolates used in this study.
