# Supplementary figures and images for: Comparative Genomic Studies of Salmonella Heidelberg Isolated From Chicken- and Turkey-Associated Farm Environmental Samples
Source: Front Microbiol. 2018 Aug 10;9:1841. doi: 10.3389/fmicb.2018.01841 (PMC6097345; doi:10.3389/fmicb.2018.01841)

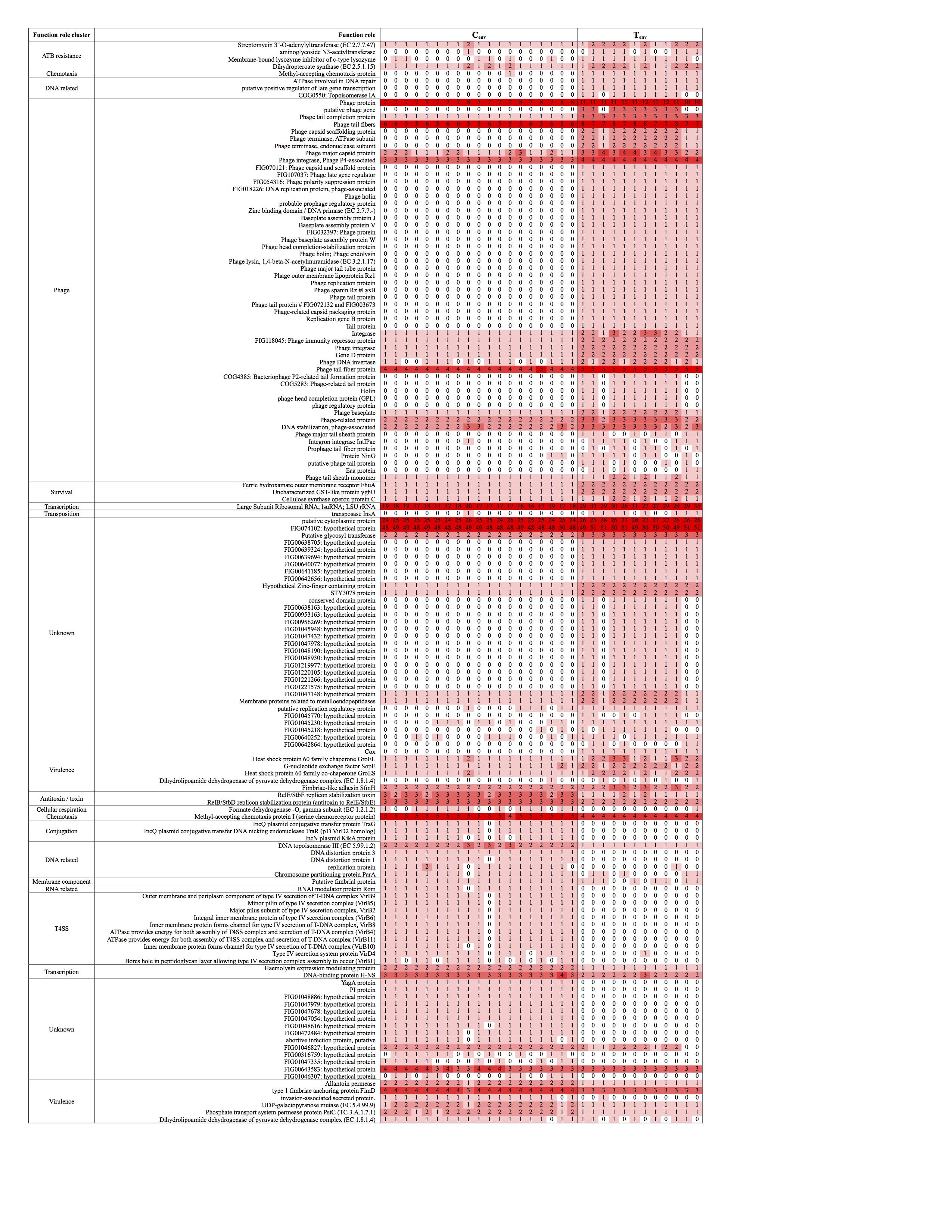

Supplement: FIGURE S1 — Heatmap of protein-encoding genes showing significant differences between production systems at the function level. Heatmap: the gradient of red is proportional to the number of protein-encoding genes detected for a specific function role, and white cells are for protein-encoding genes not detected. Numbers in each cell represent the amounts of protein-encoding genes with the designated function role. Cenv: chicken environmental isolates (n = 19); Tenv: turkey environmental isolates (n = 12). N = 152 protein-encoding genes. [file Image_1.JPEG]
