# Supplementary material for: Comparative Genomic Studies of Salmonella Heidelberg Isolated From Chicken- and Turkey-Associated Farm Environmental Samples
Source: Front Microbiol. 2018 Aug 10;9:1841. doi: 10.3389/fmicb.2018.01841 (PMC6097345; doi:10.3389/fmicb.2018.01841)
Supplement: TABLE S1 — Salmonella Heidelberg isolates used in this study. [file Table_1.docx]

**TABLE S1 | *S*. Heidelberg isolates used in this study**

| **Isolate ID** | **Sample origin** | **Sample Type** | **Production Type** | **Farm ID** | **Geographic cluster*** | **NCBI Genome accession ID** |
| --- | --- | --- | --- | --- | --- | --- |
| C_NS001 | Chicken | Environmental bootie | Broiler breeder | 1 | A | PGWO00000000 |
| C_NS002 | Chicken | Environmental bootie | Broiler breeder | 2 | A | PGWN00000000 |
| C_NS003 | Chicken | Environmental bootie | Broiler breeder | 3 | A | PGWM00000000 |
| C_NS004 | Chicken | Environmental bootie | Broiler breeder | 4 | A | PGWL00000000 |
| C_NS005 | Chicken | Environmental bootie | Broiler breeder | 5 | A | PGWK00000000 |
| C_NS006 | Chicken | Environmental bootie | Broiler breeder | 6 | A | PGWJ00000000 |
| C_NS007 | Chicken | Environmental bootie | Broiler breeder | 5 | A | PGWI00000000 |
| C_NS008 | Chicken | Environmental bootie | Broiler breeder | 7 | A | PGWH00000000 |
| C_NS009 | Chicken | Environmental bootie | Broiler breeder | 8 | B | PGWG00000000 |
| C_NS010 | Chicken | Environmental bootie | Broiler breeder | 8 | B | PGWF00000000 |
| C_NS011 | Chicken | Environmental bootie | Broiler breeder | 9 | A | PGWE00000000 |
| C_NS020 | Chicken | Environmental bootie | Broiler breeder | 8 | B | PGWD00000000 |
| C_NS024 | Chicken | Environmental bootie | Broiler breeder | 10 | A | PGWC00000000 |
| C_NS025 | Chicken | Environmental bootie | Broiler breeder | 11 | A | PGWB00000000 |
| C_NS026 | Chicken | Environmental bootie | Broiler breeder | 12 | A | PGWA00000000 |
| C_NS027 | Chicken | Environmental bootie | Broiler breeder | 13 | A | PGVZ00000000 |
| C_NS028 | Chicken | Environmental bootie | Broiler breeder | 14 | A | PGVY00000000 |
| C_NS029 | Chicken | Environmental bootie | Broiler breeder | 15 | A | PGVX00000000 |
| C_NS030 | Chicken | Environmental bootie | Broiler breeder | 16 | A | PGVW00000000 |
| T_NS-012 | Turkey | Environmental bootie | Turkey breeder | 17 | A | PGVV00000000 |
| T_NS-013 | Turkey | Environmental bootie | Turkey breeder | 18 | C | PGVU00000000 |
| T_NS-014 | Turkey | Environmental bootie | Turkey breeder | 19 | B | PGVT00000000 |
| T_NS-015 | Turkey | Environmental bootie | Turkey breeder | 20 | B | PGVS00000000 |
| T_NS-016 | Turkey | Hatchery debris | Commercial hatches | 21 | ND | PGVR00000000 |
| T_NS-017 | Turkey | Environmental bootie | Turkey breeder | 19 | B | PGVQ00000000 |
| T_NS-018 | Turkey | Environmental bootie | Turkey breeder | 22 | B | PGVP00000000 |
| T_NS-019 | Turkey | Hatchery debris | Commercial hatches | 23 | ND | PGVO00000000 |
| T_NS-031 | Turkey | Environmental bootie | Turkey breeder | 22 | B | PGVL00000000 |
| T_NS-032 | Turkey | Environmental bootie | Turkey breeder | 20 | B | PGVK00000000 |
| T_NS-033 | Turkey | Environmental bootie | Turkey breeder | 17 | A | PGVJ00000000 |
| T_NS-034 | Turkey | Environmental bootie | Turkey breeder | 24 | A | PGVI00000000 |

*: geographic clusterization was based on a multidimensional scaling combined with a Wearn links (smaller portion; cutoff= 0.33), as described in Fig 1A.

ND: not determined.
